# Supplementary material for: A de novo paradigm for male infertility
Source: Nat Commun. 2022 Jan 10;13:154. doi: 10.1038/s41467-021-27132-8 (PMC8748898; doi:10.1038/s41467-021-27132-8)
Supplement: Supplementary file 3 — Description of Additional Supplementary Files [file 41467_2021_27132_MOESM3_ESM.pdf]

File Name: Supplementary Data 1

Description: **All DNMs mutations identified in 185 patient-parent trios in NIJ/NCL Cohort of infertile men.** *Based on SIFT, MutationTaster, PolyPhen2, ACMG classification, gnomAD cases and fertile controls with variant. This score was used to initially determine the variants most likely to be damaging before moving on to the functional analysis.

File Name: Supplementary Data 2

Description**: Rare protein-coding mutations solely maternally and paternally inherited observed in 185 patient-parent trios in NIJ/NCL Cohort of infertile men.** *Based on SIFT, MutationTaster, PolyPhen2, ACMG classification, gnomAD cases; ¥ Exact mutation found in more than 1 verified fertile father; ˣMutation in same patient with DNM in TOPAZ1; DNM = De novo mutation; NIJ/NCL Cohort = Nijmegen/Newcastle Cohort.

File Name: Supplementary Data 3

Description: **Summary of rare loss-of-function (LoF) pathogenic mutations observed in additional cohorts of infertile men and fertile control cohorts.** Exome data from four additional cohorts of infertile men as well as control cohorts of fertile men and women were investigated for the presence of LoF mutations in these genes. A burden test was used to compare the total number of predicted pathogenic LoF mutations observed in the infertile vs. fertile men, as well as between fertile men and fertile women. A two-tailed Fisher's Exact test was performed with Bonferroni correction applied to adjust p-values for multiple testing of all 152 genes of interest. N/A – Data not available for this gene in WES data. Abbreviations: DNM = De novo mutation; NIJ/NCL Cohort = Nijmegen/Newcastle Cohort; GEMINI Cohort = Genetics of Male Infertility Initiative Cohort; MERGE Cohort = Male Reproductive Genomics;

File Name: Supplementary Data 4

Description: **Rare loss-of-function (LoF) pathogenic mutations observed in additional cohorts of infertile men and fertile control cohorts.**

File Name: Supplementary Data 5

Description: **Summary of rare missense pathogenic mutations observed in additional cohorts of infertile men and fertile control cohorts.** Exome data from four additional cohorts of infertile men as well as control cohorts of fertile men and women were investigated for the presence of LoF mutations in these genes. A burden test was used to compare the total number of predicted pathogenic missense mutations observed in the infertile vs. fertile men, as well as between fertile men and fertile women. A two-tailed Fisher's Exact test was performed, with Bonferroni correction was applied to adjust p-values for multiple testing of all 152 genes of interest. N/A – Data not available for this gene in WES data. Abbreviations: DNM = De novo mutation; NIJ/NCL Cohort = Nijmegen/Newcastle Cohort; GEMINI Cohort = Genetics of Male Infertility Initiative Cohort; MERGE Cohort = Male Reproductive Genomics;

File Name: Supplementary Data 6

Description: **Rare missense pathogenic mutations observed in additional cohorts of infertile men and fertile control cohorts.**

File Name: Supplementary Data 7

Description: **Clinical details of individuals with RBM5 pathogenic mutations described in this study.** Multiple infertile men from different cohorts were found with a rare pathogenic mutation in RBM5 in

addition to the Proband_108 where a DNM in RBM5 was initially identified. *At diagnosis of infertility. SCO = Sertoli cell only; N/A = Not Applicable.

File Name: Supplementary Data 8
Description: **List of primers used in Sanger Sequencing for all DNMs identified.**

File Name: Supplementary Data 9
Description: **Clinical data of Patients in NIJ/NCL Cohort of infertile Men.**

File Name: Supplementary Data 10
Description: **Treatment outcome for Patients in NIJ/NCL Cohort of infertile Men.**

File Name: Supplementary Data 11
Description**: Definition of terms used in the histology classification of patients.**

File Name: Supplementary Data 12
Description: **Pathology notes on Patients in NIJ/NCL Cohort of infertile Men.**

File Name: Supplementary Data 13
Description: **Cytology data from Patients in NIJ/NCL Cohort of infertile Men.**
