# Supplementary material for: A de novo paradigm for male infertility
Source: Nat Commun. 2022 Jan 10;13:154. doi: 10.1038/s41467-021-27132-8 (PMC8748898; doi:10.1038/s41467-021-27132-8)
Supplement: Supplementary file 5 — Reporting Summary [file 41467_2021_27132_MOESM5_ESM.pdf]

nature portfolio

Corresponding author(s): Joris Veltman

Last updated by author(s): Oct 7, 2021

Reporting Summary

Nature Portfolio wishes to improve the reproducibility of the work that we publish. This form provides structure for consistency and transparency in reporting. For further information on Nature Portfolio policies, see our Editorial Policies and the Editorial Policy Checklist.

Statistics

For all statistical analyses, confirm that the following items are present in the figure legend, table legend, main text, or Methods section.

n/a	Confirmed	
☐	☒	The exact sample size (*n*) for each experimental group/condition, given as a discrete number and unit of measurement
☐	☒	A statement on whether measurements were taken from distinct samples or whether the same sample was measured repeatedly
☐	☒	The statistical test(s) used AND whether they are one- or two-sided<br>*Only common tests should be described solely by name; describe more complex techniques in the Methods section.*
☐	☒	A description of all covariates tested
☐	☒	A description of any assumptions or corrections, such as tests of normality and adjustment for multiple comparisons
☐	☒	A full description of the statistical parameters including central tendency (e.g. means) or other basic estimates (e.g. regression coefficient) AND variation (e.g. standard deviation) or associated estimates of uncertainty (e.g. confidence intervals)
☐	☒	For null hypothesis testing, the test statistic (e.g. *F*, *t*, *r*) with confidence intervals, effect sizes, degrees of freedom and *P* value noted<br>*Give P values as exact values whenever suitable.*
☒	☐	For Bayesian analysis, information on the choice of priors and Markov chain Monte Carlo settings
☒	☐	For hierarchical and complex designs, identification of the appropriate level for tests and full reporting of outcomes
☒	☐	Estimates of effect sizes (e.g. Cohen's *d*, Pearson's *r*), indicating how they were calculated

Our web collection on statistics for biologists contains articles on many of the points above.

Software and code

Policy information about availability of computer code

Data collection	Sequencing data was generated from DNA samples prepared Illumina's Nextera DNA Exome Capture kit or Twist Bioscience's Twist Human Core Exome Kit seqenced on the NovaSeq 6000 Sequencing System (Illumina). Validation of de novo SNP variants was performed using Sanger sequencing on an Applied Biosystems SeqStudio Genetic Analyzer (ThermoFisher). Validation of CNVs was performed using the whole genome Illumina Infinium CytoSNP-850 v1.1 microarray platform (Illumina) and using the Applied Biosystems QuantStudio 7 Flex Real-Time PCR System (ThermoFisher). Long-range sequencing was achieved using Oxford Nanopore's MinION sequencer (Oxford Nanopore Technologies).
Data analysis	Data processing and analysis was conducted following best practices using BWA Mem v0.7.17, Picard v2.0.1, GATK v4.1.4.1. Annotation of data was obtain via Ensemble's VEP v99 and AnnotSV 2.2. Protein-protein interaction analysis were performed using STRING v11. Primers for variant validation and for targeted long-read sequencing were designed with Primer3 v2.3.6. Long-read sequencing generated was processed using Guppy v3.4.4, cutadapt v2.5, BWA Men v0.7.17 and SAMtools v0.1.19. Statistical analysis was performed in R v4.0.2.

For manuscripts utilizing custom algorithms or software that are central to the research but not yet described in published literature, software must be made available to editors and reviewers. We strongly encourage code deposition in a community repository (e.g. GitHub). See the Nature Portfolio guidelines for submitting code & software for further information.

Data

Policy information about availability of data

All manuscripts must include a data availability statement. This statement should provide the following information, where applicable:

- Accession codes, unique identifiers, or web links for publicly available datasets
- A description of any restrictions on data availability
- For clinical datasets or third party data, please ensure that the statement adheres to our policy

Sequencing data have been deposited in the European Genome-phenome Archive(EGA) under the accession code EGAS00001005417 and will be made available upon reasonable request for academic use and within the limitations of the provided informed consent by the corresponding author upon acceptance. Every request will be reviewed by the Newcastle University Male Infertility Genomics Data Access Committee; the researcher will need to sign a data access agreement after approval.

Field-specific reporting

Please select the one below that is the best fit for your research. If you are not sure, read the appropriate sections before making your selection.

☒ Life sciences ☐ Behavioural & social sciences ☐ Ecological, evolutionary & environmental sciences

For a reference copy of the document with all sections, see nature.com/documents/nr-reporting-summary-flat.pdf

Life sciences study design

All studies must disclose on these points even when the disclosure is negative.

Sample size	Power calculation for the identification of candidate genes and/or genomic regions of interest from de novo mutations in patient-parent trio based on the prior similar studies with patient-parent cohorts (Gilissen et al. Nature 2014) estimate that 500 trios are required for a full strength study but that a the lower end significant results can be found using 50 patient-parent trios. Due to the nature of the disorder being investigated and difficulties associated with the recruitment of the parents of patients in their 30s and 40s, affected by a disease that remain surrounded by societal stigma and taboos, we were forced to conduct our analysis on a smaller sample size that still permits significant conclusions to be drawn. To this end a total of 185 patients who presented with unexplained (idiopathic) azoospermia (N=111) or severe to extreme oligozoospermia (with or without asthenozoospermia N=74) at the Radboudumc outpatient clinic between July 2007 and October 2017 (N=170) and at the Newcastle upon Tyne Hospitals NHS Foundation Trust (Newcastle, UK) between January 2018 to January 2020 (n=15) were used in this study.
Data exclusions	Patients where clinical evaluation lead to an etiologic diagnosis and/or were found to carry AZF deletions or chromosomal anomalies were excluded from this study. Patients that were found to not have 2 semen analysis conclusively indicating have no or reduced numbers of spermatozoa consistent with azoospermic and severe oligozoospermic classifications were no included in the study. Patients for which one or both biologicals parents could not be contacted or did not wish to participate in the study were excluded from this study. Detailed information for inclusion and exclusion parameters is present in supplementary notes. Exome sequencing data from a single member of a trio, either the patient or one of parents, not reaching the minimum threshold of 30X average coverage, more than 50% of sequenced reads above 30X and 30 million reads, was sufficient to exclude the entire trio from being included in this study.
Replication	Exact replication is not achievable at this stage with no other cohort of patient-parent trios for this disorder of similar size and composition worldwide. Patients presenting identical characteristics were used for comparison as detailed in the main article and methods.
Randomization	Patients were allocated to experimental groups according to the conclusions of a thorough clinical examination to their fertility status. Fertile samples were obtained from confirmed biological parents.
Blinding	Blinding of participants during recruitment was conducted by clinicians approaching all patients presenting at the clinics displaying fertility issues. Semen analyses and clinical examinations were performed blind by the technicians and clinicians to the patient's fertility status since in only 50% of the couples affected by fertility issues are due to male infertility. The clinical diagnosis and health care recommendations to the couples of the men participating in this study was due independently of their final inclusion or exclusion from the study cohort. Due to the study design it was essential that that fertile parents were analysed together with their respective infertile sons to this purpose blinding was not kept when performing exome sequencing or bioinformatic processing of the resulting data. However, blinding of the phenotype associated with each patient was kept during genetic analysis and interpretation of de novo mutations, only after was the exact phenotype of the patients revealed to assess the likelihood of the genetic variants being the cause of the presented phenotype.

Reporting for specific materials, systems and methods

We require information from authors about some types of materials, experimental systems and methods used in many studies. Here, indicate whether each material, system or method listed is relevant to your study. If you are not sure if a list item applies to your research, read the appropriate section before selecting a response.

Materials & experimental systems

n/a	Involved in the study
☐	☒ Antibodies
☒	☐ Eukaryotic cell lines
☒	☐ Palaeontology and archaeology
☒	☐ Animals and other organisms
☐	☒ Human research participants
☒	☐ Clinical data
☒	☐ Dual use research of concern

Methods

n/a	Involved in the study
☒	☐ ChIP-seq
☒	☐ Flow cytometry
☒	☐ MRI-based neuroimaging

Antibodies

Antibodies used	One antibody was used in this study: anti-RBM5, supplier: Atlas Antibodies, Cat. nr.: HPA018011.
Validation	Antibody was extensively validated by manufacturer with Western blotting, masspectometry and protein arrays. Link to antibody website: https://www.proteinatlas.org/ENSG00000003756-RBM5/antibody

Human research participants

Policy information about studies involving human research participants

Population characteristics	Patients were adult males (20 to 49 years of age) presenting at the fertility clinics after unsuccessfully achieving conception with their partners for which clinical evaluations did not reveal an etiologic diagnosis and were negative for AZF deletions and chromosomal anomalies. The parents of the recruited patients aged 35-75 years were verified as the biological progenitors of each the patient and consequently fertile at the time of conception. Individuals were recruited at the Netherlands and the United Kingdom with the majority of individuals being of European (Dutch or British) decent, with one family from Arabic descent, another from the Caribbean Islands and 4 of undermined genetic descent. The defining characteristics of the patients in this study was their semen parameters with 111 diagnosed as azoospermic and 74 as oligozoospermic.
Recruitment	Participants were recruited from male patients who presented with idiopathic azoospermia or severe to extreme oligozoospermia (with or without asthenozoospermia) at the Radboudumc outpatient clinic (The Netherlands) between July 2007 and October 2017 and at the Newcastle upon Tyne Hospitals NHS Foundation Trust (Newcastle, UK). By performing the recruitment at select clinics alone produced a recruitment bias that excludes infertile individuals not attending these or any clinics, nevertheless couples attending these clinics were found to cover most social and economic backgrounds in these countries. The bias of recruiting a predominately European population means that the conclusions of this study may only apply to an overall European population and not be representative of other human populations.
Ethics oversight	The study protocol was approved by the respective Ethics Committees/Institutional Review Boards (Nijmegen: NL50495.091.14 version 4, Newcastle: REC Ref: 18/NE/0089) and written informed consent from all patients and their parents was obtained.

Note that full information on the approval of the study protocol must also be provided in the manuscript.
